# Replantation of lamina spinous process ligament complex and miniature titanium plate shaping internal fixation in the treatment of tumors in the spinal canal

**DOI:** 10.1186/s12891-023-06984-9

**Published:** 2023-11-10

**Authors:** Guohua Dai, Min Zhang, Weiliang Su, Jiaqing Zhao, Xiankai Yu, Zhaozhong Sun, Yongfeng Dou, Xiaopeng Geng

**Affiliations:** Department of Spine Surgery, Binzhou Medical University Hospital, 661 Second Huanghe road, Bincheng District, Binzhou, Shandong Province 256600 China

**Keywords:** Microporous titanium plate, Reimplantation of lamina spinous process ligament complex, Intraspinal Tumor, Thoracolumbar spine

## Abstract

**Objective:**

Purpose This study aims to explore the clinical efficacy of laminospinous process ligament complex reimplantation combined with mini-titanium plate fixation in the treatment of thoracolumbar intraspinal tumors.

**Methods:**

A retrospective analysis was performed on 43 cases of intraspinal tumors treated with thoracolumbar intraspinal tumor resection from August 2018 to March 2021, and 27 cases underwent laminospinous process ligament complex reimplantation combined with micro titanium plate shaping. Fixation (laminar replantation group), and 16 patients underwent laminectomy combined with pedicle screw internal fixation (laminectomy group). The operation time, blood loss, drainage tube removal time, cerebrospinal fluid leakage, spinal instability, and the incidence of secondary spinal stenosis were compared between the two groups. The pain VAS score, ODI score, and modified Macnab at the last follow-up were compared between the two groups. And the laminar fusion rate of the laminoplasty group was measured.

**Results:**

Both groups successfully completed the surgery and obtained complete follow-up. The incidence of cerebrospinal fluid leakage and secondary spinal canal stenosis in the laminectomy group was lower than that in the laminectomy group, and the difference was statistically significant (*P* < 0.05). There was no statistically significant difference in the incidence of spinal instability between the two groups (*P* > 0.05). The operation time and intraoperative blood loss in the laminectomy group were less than those in the laminectomy group, and the drainage tube removal time was earlier than that in the laminectomy group. The difference was statistically significant (*P* < 0.05). At the final follow-up, there was no statistically significant difference in the pain VAS score, ODI score, and modified Macnab between the two groups (*P* > 0.05), but they were all significantly improved compared with preoperative ones. Fusion evaluation was conducted on the laminoplasty group. Two years after surgery, the fusion rate was 97.56% (40/41).

**Conclusions:**

The application of laminospinous process ligament complex reimplantation combined with mini titanium plate fixation during thoracolumbar intraspinal tumor resection can effectively reconstruct the spinal canal and posterior column structure, reduce the incidence of cerebrospinal fluid leakage and secondary spinal stenosis. The laminar fusion rate is high.

## Introduction

Primary intraspinal tumors account for 2–15% of all central nervous system (CNS) tumors [1]. Most intraspinal tumors are benign, and about 40% of them are intradural [2], and early surgery is the treatment of choice [3]. Surgical methods usually include: total laminectomy, hemilaminectomy, laminectomy combined with pedicle fixation, laminoplasty combined with titanium plate fixation, etc. [1, 4, 5]. The common point of the above surgical methods is to completely remove the tumor in the spinal canal and fully decompress the spinal nerves. The difference is that the treatment methods for the stability of the spine are different. The basic principle of intraspinal tumor surgery is to completely resect the tumor and restore the stability of the spine [6]. Laminectomy is to achieve tumor exposure and resection, but it also brings some complications, such as epidural scar adhesion, spinal instability, kyphosis, etc. [7]. At present, more and more spine surgeons are aware of the importance of reconstructing spinal stability for postoperative recovery and improving the quality of life of patients [8]. Following the introduction of laminar replantation by Raimondi in 1976 [9], various laminoplasty procedures were used for tumor resection in the spinal canal [6, 8, 10–13].

## Information and methods

In this study, patients who underwent surgical treatment for thoracolumbar intracanal tumors from August 2018 to March 2021 were retrospectively collected. Inclusion criteria: (1) all patients received surgical treatment for the first time; (2) clinical and pathological data were complete; (3) follow-up time was greater than 2 years. Exclusion criteria: (1) Tumor causes spinal structural destruction or spinal instability.

## Patient information

We included 43 patients with thoracolumbar intraspinal tumors. All patients were diagnosed by lumbar spine X-ray, computed tomography (CT), and magnetic resonance imaging (MRI) before surgery. After the lesion is discovered in a routine scan, an enhanced scan is performed to clarify the scope and size of the tumor and its relationship with the spinal cord, cauda equina, and nerve roots. This study was conducted in accordance with the Declaration of Helsinki and approved by the Ethics Committee of Binzhou Medical College Affiliated Hospital. Written informed consent was obtained from all participants.

## Surgical technique (laminar replantation group)

Anesthesia and Positioning. All patients were under general anesthesia with endotracheal intubation and operated in prone position.

## Method and exposure

**Fig. 1:**
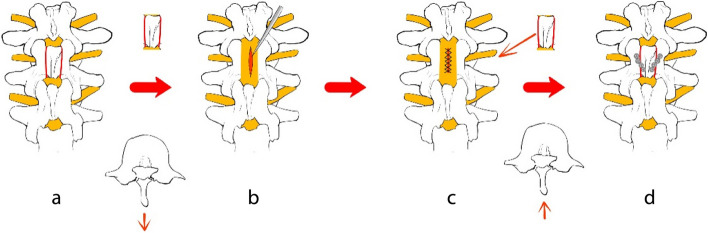
The process of removing and replanting vertebral laminae spinous ligament complex

 Through the midline posterior approach, the paraspinal muscles are detached from the spinous process and the lamina, and the supraspinous ligament and interspinous ligament are preserved. Then, a minimal laminectomy is performed between the sides of the spinous process and the inner edge of the facet joint with an ultrasonic osteotome surgery. The supraspinous and interspinous ligaments of the head and caudal segments of the lamina were cut, and subsequently, the free spinous process-lamellar complex was removed with forceps and placed on a workbench outside the surgical field (Fig. 1a).

## Tumor resection

Under the microscope, a longitudinal incision was made in the posterior center of the dura mater, and it was pulled apart with a silk thread. The tumor was completely dissected and resected, decompressing the spinal cord (Fig. 1b). Waterproof sutures were applied to the dura after tumor resection (Fig. 1c).

## Reconstruction of the posterior column

Micro titanium plates are molded for an optimal fit. The shaped titanium plates were fixed on the lamina on both sides with screws (Fig. 1d). Finally, the supraspinous ligaments cranial and caudal to the lamina were secured with tendon sutures to further maintain the in situ reimplantation of the spinous process-laminar complex. Drainage tubes were placed, the incision was flushed, and sutured layer by layer.

## Surgical technique (laminectomy group)

Routinely expose the lamina to the outer edges of the facet joints on both sides, insert pedicle screws at the corresponding segments, and completely remove the lamina from the isthmus upward along the inner edge of the superior facet joints. The tumor resection method is the same as above, and a connecting rod is installed and passed posterolaterally. Bone graft fusion.

## Postoperative managem

Removing the drainage tube when the drainage rate is <50 mL.Guiding the patient after removing the drainage tube Bed back muscle function exercise.Geting out of bed with waist protection, use antibiotics prophylactically after operation, and wear waist circumference for 1 month after operation.

## Result evaluation

### Treatment assessment

All patients were followed for at least 24 months. The data were recorded, and the operation time, blood loss, drainage tube removal time, and incidence of cerebrospinal fluid leakage, spinal instability, and secondary spinal stenosis were compared between the two groups. The pain visual analogue score (VAS) was used to evaluate the degree of low back and leg pain, the Oswestry disability index (ODI) was used to evaluate the functional improvement, and the modified Macnab was used to evaluate the clinical efficacy at the final follow-up. If the difference in the Cobb angle of the injured vertebra at the surgical segment in the lateral hyperextension and hyperflexion X-rays is smaller than the preoperative value, it indicates spinal movement dysfunction. X-ray films 6 months after surgery show that the Cobb angle of the injured vertebrae at the surgical segment is greater than the normal physiological angle or intervertebral body slippage occurs, indicating spinal instability; the cross-section of the MRI film measures the transverse diameter of the dural sac at the narrowest point of the surgical segment. and deformed diameter, if the sum of the two is less than the preoperative level, it is secondary spinal stenosis. The lamina replantation group underwent lumbar spine CT scans at 6, 12, and 24 months after surgery to evaluate the bone fusion. If there was a bone bridge on one side or on both sides, it was defined as “bone fusion” Six months after the operation, MRI examination showed no recurrence of the tumor.

### Statistical analysis

The SPSS 24.0 (SPSS Company, USA) statistical software package was used for statistical analysis and processing of the data. The measurement data were tested for normality, expressed as t test, and repeated measures analysis of variance were used to compare the two groups at different time points. Count data were compared using the χ2 test. *P* < 0.05 means the difference is statistically significant.

## Results

Both groups successfully completed the surgery and received complete follow-up for 24–36 months (27.3 ± 1.5). During the follow-up period, there was no tumor recurrence, neurological damage, or surgical incision infection. The incidence of cerebrospinal fluid leakage, operation time, blood loss, drainage tube removal time, and incidence of spinal motor dysfunction in the laminectomy group were all lower than those in the laminectomy group, and the differences were statistically significant (*P* < 0.05). The incidence of spinal instability in the laminectomy group was lower than that in the laminectomy group, and the difference was not statistically significant. There was no statistically significant difference in pain VAS score, ODI score, and modified Macnab between the two groups at the final follow-up (*P* > 0.05); however, the postoperative VAS score and ODI score were significantly improved compared with those before surgery, with statistical significance (*P* < 0.05). (See Tables 1 and 2 for details). A total of 41 segments of laminoplasty were successfully performed on 27 patients in the laminoplasty group. There were 13 cases of single segment and 14 cases of 2 segments. At the last follow-up, the “fusion” rate was 97.56% (40/41).

**Table 1:** Comparison of perioperative data between the two groups

	Laminectomy group (*n* = 16)	Laminoplasty group (*n*=27)	*P*
Operation time (min)	161.75 ± 4	134.93 ± 6.5	≤ 0.05
Intraoperative blood loss (ml)	222.69 ± 4.5	155.85 ± 1.5	≤ 0.05
Cerebrospinal fluid (CSF) leakage	10	4	≤ 0.05
Drainage tube removal time (day)	6 ± 0.5	3.96 ± 0.5	≤ 0.05
Incidence of spinal instability	1	1	> 0.05
Incidence of spinal motor dysfunction	1	1	> 0.05

**Table 2:** Comparisons of VAS scores and ODI at different time points before and after operation between groups

	Preoperative		Postoperative 3 months		Last follow-up	
	Laminectomy group (*n*=16)	Laminoplasty group (*n*=27)	Laminectomy group (*n*=16)	Laminoplasty group (*n*=27)	Laminectomy group (*n*=16)	Laminoplasty group (*n*=27)
VAS	7.75±0.5	7.44±0.5	2.25±0.5	1.93±0.5	1±0.5	0.81±0.5
ODI	75.69%±0.5%	77.81%±3.5%	19.31%±3%	21.63%±6%	10.81%±1%	12.04%±3%

### Illustrative case

**Fig. 2:**
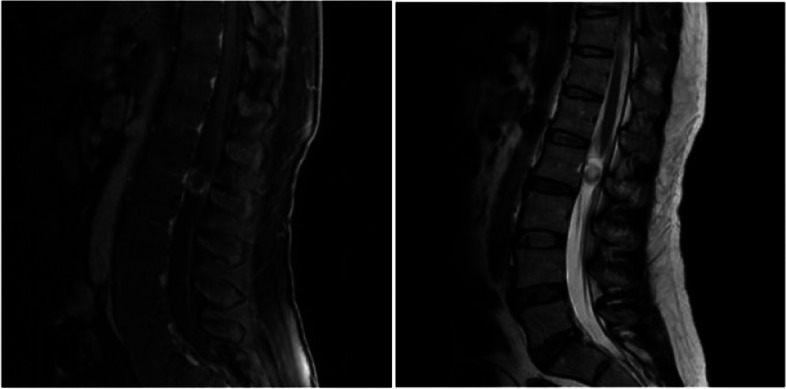
Preoperative lumbar MRI showed intraspinal space occupying lesions at L2 level

**Fig. 3:**
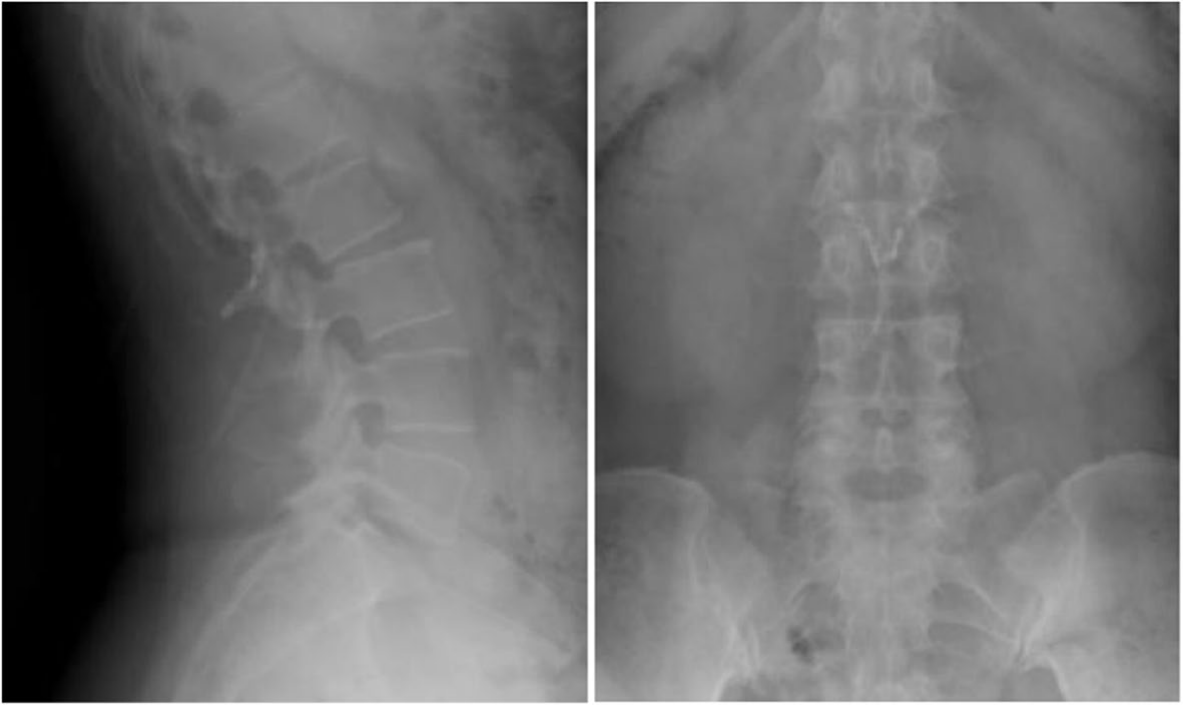
One month after operation, the positive X-ray plain film of lumbar showed that the replanted lamina began to heal

**Fig. 4:**
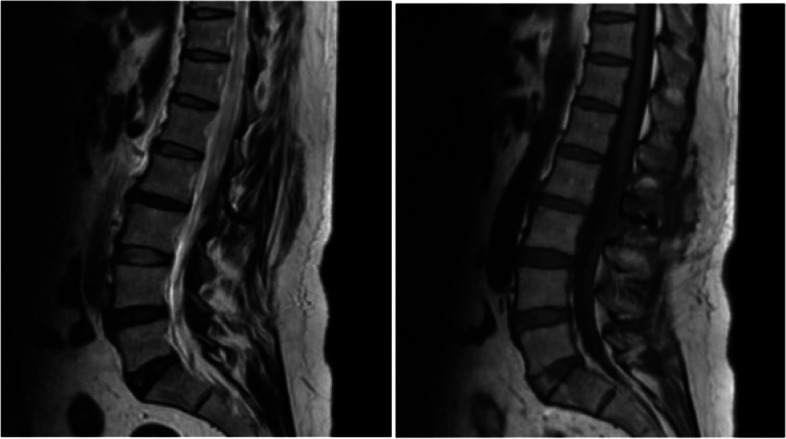
Six months after operation, lumbar MRI showed that the ligaments were repaired and healed, and there was no intraspinal scar adhesions or restenosis

**Fig. 5:**
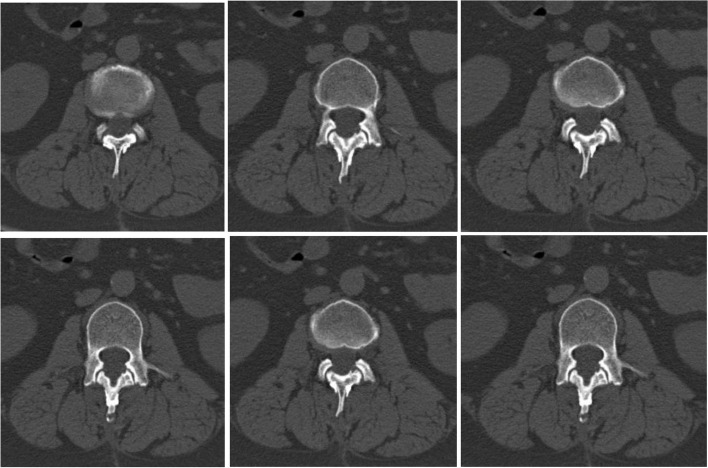
Twenty-four months after operation, the replanted lamina was almost completely fused

**Fig. 6:**
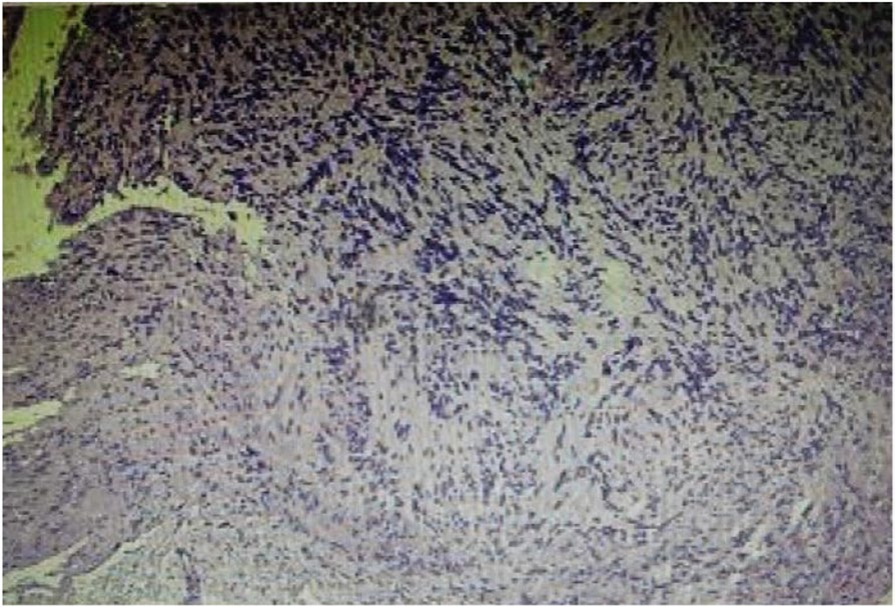
HE 10 × 40 Postoperative pathological findings: schwannoma

The patient, a 38-year-old female, suffered from pain and numbness in both lower limbs for 6 years, which worsened for 15 days. MRI of the lumbar spine showed space-occupying lesions in the spinal canal. Physical examination on admission: mild tenderness in the lumbar spinous process. The skin sensation in both calves and feet is reduced. The preoperative VAS score was 7 points and the ODI score was 74%. Laminoplasty combined with mini-titanium plate shaping to treat intraspinal tumors at L2 level. Histological examination of the surgical specimen confirmed the diagnosis of schwannoma. The pain in the lower limbs disappeared after the operation, and the numbness of both lower limbs was significantly relieved. There were no complications such as cerebrospinal fluid leakage. One month after surgery, the VAS score was 2 points and the ODI score was 25%. Six months after surgery, no tumor recurrence was found on lumbar spine MRI. Twelve months after surgery, the VAS score was 0 and the ODI score was 5%. At the same time, the lower limb function returned to normal, and CT showed that the transplanted lamina was completely healed (Figs. 2, 3, 4, 5 and 6).

Clinical imaging from one representative patient (female, 38 years old) more than 12 months after Replantation of lamina spinous process ligament complex and miniature titanium plate shaping internal fixation (L2) (Fig.  2). Preoperative lumbar MRI showed intraspinal space occupying lesions at L2 level (Fig. 3). One month after operation, the positive X-ray plain film of lumbar showed that the replanted lamina began to heal (Fig. 4). Six months after operation, lumbar MRI showed that the ligaments were repaired and healed, and there was no intraspinal scar adhesions or restenosis (Fig. 5). 24 months after operation, the replanted lamina was almost completely fused (Fig. 6). (HE 10×40 Postoperative pathological findings: schwannoma).

## Discussion

### Rebuild biomechanical stability

Although the traditional tumor resection in the spinal canal completely removed the tumor and relieved the compression of the spinal nerve, it also brought some problems, such as chronic low back pain caused by lumbar instability [6], epidural scar adhesion caused spinal canal stenosis, difficult revision of tumor recurrence, kyphosis, etc. [11, 14]. More and more scholars realize that the reconstruction of spinal stability is as important as the resection of tumors in the spinal canal. Based on Denis’ “three-column theory” [15], the biomechanical stability of the spine depends on the integrity of the anatomical structure of the spine, and the replantation of the spinous process laminar ligament complex is conducive to the recovery of the anatomical structure and function of the spine [1, 8], so as to realize the unification of structure and function.

### Laminar reimplantation and titanium plate internal fixation

Surgical methods include total laminectomy, hemilaminectomy, laminectomy combined with pedicle internal fixation, laminectomy and titanium plate internal fixation [1, 4, 5]. Both total laminectomy and hemilaminectomy cause certain damage to the stability of the spine [6]. Traditional surgical suture fixation, steel wire fixation, and pedicle screw fixation can easily lead to lamina displacement and bone insufficiency. The fusion rate of joints and bones is low [16, 17]. Laminectomy and pedicle screw internal fixation will lead to the loss of motion function of the corresponding segments, accelerate degenerative changes in the spine, and even induce adjacent vertebral body lesions [18]. Scar contracture may also cause compression of the dural sac, and even Symptoms of nerve compression occur [19]. In order to avoid the above situation and simultaneously achieve the goal of opening the spinal canal to remove the tumor while avoiding the destruction of the posterior column structure of the spine leading to the loss of motion function of the corresponding segment, new surgical methods are urgently needed, namely, lamina reimplantation and titanium plate internalization. Immobilization (laminoplasty) [20]. Compared with traditional surgery, laminoplasty can better maintain spinal stability, preserve spinal mobility, and reduce postoperative complications [1]. After tumor resection, replantation of the spinous process-laminar ligament complex is beneficial to restoring the anatomical structure and function of the spine and reducing postoperative complications, such as kyphosis and iatrogenic spinal stenosis. At the same time, the restoration of the ligament-neuro-muscle reflex system of the supraspinal and interspinous ligaments contributes to the movement of the lower back. In addition, considering the possible recurrence of intradural tumors, the relatively normal posterior skeletal structure and the absence of epidurals are retained. scar, making revision surgery easier and safer [8]. At the same time, biomechanical tests have shown that laminoplasty combined with micro-titanium plate fixation can improve the stability, compression resistance, and resistance to bending, shearing, and rotation of the spine [6]. Laminoplasty is gradually becoming an increasingly popular surgical procedure, and its advantages are gradually becoming apparent in clinical application [14]. In this study, the shaped micro titanium plate was used to increase the contact area between the complex and the vertebral plate, achieve perfect fit, improve the bone fusion rate, and at the same time reduce the use of micro titanium plates and reduce surgical costs. In the laminoplasty group, only the inner side of the articular process is exposed during the operation. There is no need to expose it too much, and there is no need to use pedicle screws for internal fixation.The operation time is short, the intraoperative blood loss is small, and the spinal mobility can be better preserved. After spinal canal reconstruction, the incidence of cerebrospinal fluid leakage is reduced, the postoperative drainage volume is reduced, and the postoperative drainage tube removal time is advanced; after lamina replantation, it can By blocking the blood from the incision outside the spinal canal, the probability of hematoma forming in the spinal canal is reduced, epidural scars will be significantly reduced in the later period, and the spinal canal volume will be maintained after surgery.

### Limitations

This study is a retrospective study with a small sample size and no longer-term follow-up. The clinical efficacy remains to be further observed.

## Data Availability

The datasets used and analysed during the current study are available from the corresponding author on reasonable request.

## Abbreviations

VAS: Visual analog scale
ODI: Oswestry Disability Index
CT: Computed tomography
MRI: Magnetic resonance imaging
